# Lack of cathepsin activities alter or prevent the development of lung granulomas in a mouse model of sarcoidosis

**DOI:** 10.1186/1465-9921-12-13

**Published:** 2011-01-20

**Authors:** Andriy O Samokhin, Jacques Yves Gauthier, M David Percival, Dieter Brömme

**Affiliations:** 1 Department of Oral Biological and Medical Sciences, Faculty of Dentistry, University of British Columbia, Vancouver, BC, Canada; 2 Merck Frosst Center for Therapeutic Research, Kirkland, QC, Canada

## Abstract

**Background:**

Remodeling of lung tissues during the process of granuloma formation requires significant restructuring of the extra-cellular matrix and cathepsins K, L and S are among the strongest extra-cellular matrix degrading enzymes. Cathepsin K is highly expressed in various pathological granulomatous infiltrates and all three enzymes in their active form are detected in bronchoalveolar lavage fluids from patients with sarcoidosis. Granulomatous inflammation is driven by T-cell response and cathepsins S and L are actively involved in the regulation of antigen presentation and T-cell selection. Here, we show that the disruption of the activities of cathepsins K, L, or S affects the development of lung granulomas in a mouse model of sarcoidosis.

**Methods:**

Apolipoprotein E-deficient mice lacking cathepsin K or L were fed Paigen diet for 16 weeks and lungs were analyzed and compared with their cathepsin-expressing littermates. The role of cathepsin S in the development of granulomas was evaluated using mice treated for 8 weeks with a potent and selective cathepsin S inhibitor.

**Results:**

When compared to wild-type litters, more cathepsin K-deficient mice had lung granulomas, but individually affected mice developed smaller granulomas that were present in lower numbers. The absence of cathepsin K increased the number of multinucleated giant cells and the collagen content in granulomas. Cathepsin L deficiency resulted in decreased size and number of lung granulomas. *Apoe-/- *mice treated with a selective cathepsin S inhibitor did not develop lung granulomas and only individual epithelioid cells were observed.

**Conclusions:**

Cathepsin K deficiency affected mostly the occurrence and composition of lung granulomas, whereas cathepsin L deficiency significantly reduced their number and cathepsin S inhibition prevented the formation of granulomas.

## Background

Cathepsins K, L and S are members of the papain family of cysteine proteases that have been recently implicated in the development of various lung diseases [1]. It is believed that cathepsins participate in lung tissue remodeling based on their ability to degrade extra-cellular matrix, their involvement in the regulation of immune responses and their potential contribution to the kallikrein-kinin system [2-5]. In this study, we investigated the roles of cathepsins K, L, and S in the development of granulomas in mouse lungs that have features of human granulomas of sarcoidosis [6]. Sarcoidosis is a systemic disease characterized by the presence of noncaseating epithelioid granulomas with a pulmonary involvement in ~90% of the patients [7,8]. Cathepsins K, L, and S are expressed by lung macrophages, fibroblasts, and epithelial cells [9]. Significant expression of cathepsin K was first found in granulomas induced by Echinococcus granulosus in bovine lung [10]. A strong expression of cathepsin K was also observed in epithelioid and multinucleated giant cells (MGCs) in patients with sarcoidosis and tuberculosis [11,12]. The presence of active forms of cathepsins K, L and S was found in bronchoalveolar lavage fluids from patients with sarcoidosis [13]. Recently, we described that *Apoe-/- *mice fed a cholate-containing high fat diet develop lung granulomas that have many features of human granulomas of sarcoidosis [6]. Epithelioid and MGCs in such granulomas have shown strong immunostaining for cathepsin K suggesting that this protease might be involved in granuloma formation or resorption (6).

Similarly to cathepsin K, cathepsins L and S are able to degrade major extracellular proteins [14] but they are also involved in immune responses [15,16]. Since cathepsins L and S play significant role in antigen presentation and T cell selection [15,17] and the formation of granulomas has been linked to T cell activation [18-21], the disruption of cathepsins L and S activities might affect the development of granulomas.

Thus, based on the strong expression of cathepsin K in granulomas and the pivotal role of cathepsins S and L in the antigen presentation and T-cell selection, we hypothesized that the disruption of these protease activities might interfere with lung granuloma formation. In this study, we investigated the effect of cathepsin K and L deficiencies and cathepsin S inhibition on the development of lung granulomas in *Apoe-/- *mice on Paigen diet.

## Methods

### Animals

Three groups of mice were used: apolipoprotein E-deficient (*Apoe-/-*) mice (Jackson Laboratories), double knockout mice lacking apolipoprotein E and cathepsin K (*Apoe-/- Ctsk-/-*) (n = 10) and double knockout mice lacking apolipoprotein E and cathepsin L (*Apoe-/- Ctsl-/-*) (n = 19). Double knockout *Apoe-/- Ctsk-/- *and *Apoe-/- Ctsl-/- *mice were generated by crossing *Apoe-/- *with the *Ctsk-/- *or *Ctsl-/- *mice as previously described [22]. *Ctsk-/- *and *Ctsl-/- *mice were kindly provided by Drs. P. Saftig (University of Kiel, Germany) and C. Peters (University of Freiburg, Germany). The single cathepsin deficiencies have the following phenotypes: Cathepsin K-deficient mice show an osteopetrotic phenotype with excessive trabeculation of the bone-marrow space [23] and increased fibrosis in lungs after treatment with bleomycin [24]. Cathepsin L-deficient mice deficient mice develop periodic hair loss and epidermal hyperplasia [25].

**Figure 1:**
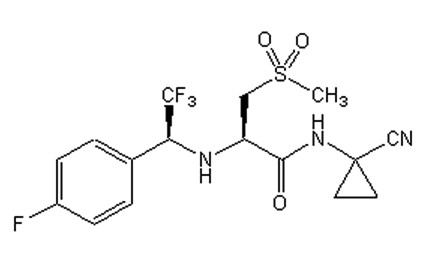
**Structure of the cathepsin S inhibitor**. Nitrile-based cathepsin S inhibitor (compound 7 according to [26]).

The following primer sequences were used for ApoE: 5"-GCCTAGCCGAGGGAGAGCCG-3"; 5"-TGTGAC TTGGGAGCTCTGCAGC-3"; 5"-GCCGCCCCGACTGCATCT-3"; for cathepsin K: 5"-GCCACACCCACACCCTAGAAG-3"; 5"-ACA AGT GTA CAT TCC CGT ACC-3"; and for cathepsin L: 5"-GGAGGGAGAGCGATATGGG-3"; 5"-TTCCTCATTGGTCTTCCG-3"; 5"-CGGAGAACCTGCGTGCAATCC-3". At 6 weeks of age mice were switched from normal to Paigen fat diet (HFD) which contained 18% fat, 1% cholesterol and 0.5% cholate; Test diet 57W4; Purina Mills. LLC, St. Louis, MO). After 16 weeks on HFD, mice were euthanized after overnight fasting by exsanguination under xylazine-ketamine anesthesia. Another two groups of *Apoe-/- *mice were studied after 8 weeks on HFD, one group received high fat diet alone (n = 12) and another received the same diet plus 0.01% w/w cathepsin S inhibitor (n = 12). The medicated diet was formulated by mixing the chow in meal form with finely dispersed crystalline, orally-active inhibitor to obtain a uniform distribution. The cathepsin S inhibitor (cpd 6 in [26]) was dosed as the sulfoxide prodrug (cpd 7 (Figure 1) in [26]) which is rapidly converted into the active sulfone inhibitor *in vivo*. The mice consumed approximately 4 g food per day with no difference in food intake or body weight increase (average final body weight 23.7 g males, 20.0 g females) between the two groups. Control *Apoe-/- *mice used for CD4^+ ^cell quantification in lungs were on normal diet for 22 weeks. Single cathepsin K and L null mice on high fat diet were not evaluated as previous experiments revealed that the induction of lung granuloma formation by high fat diet was dependent on the apolipoprotein E deficiency [6]. All animal procedures were approved by the Canadian Council on Animal Care.

### Tissue preparation

After perfusion with ice-cold phosphate-buffered saline (PBS), lungs and thymuses were fixed in 10% buffered formalin for 5 hours at 4°C, embedded in paraffin, and 5 μm sections were cut and mounted onto Superfrost/Plus slides (Fisher-Scientific, Ottawa, ON).

### Immunohistochemistry

Sections were dewaxed, rehydrated, blocked with 10% goat serum in PBS, and incubated overnight at 4°C with the primary antibody in 1% BSA in PBS: affinity purified rabbit polyclonal anti-cathepsin K (1: 50) [27], mouse monoclonal antibody against SMC α-actin (1:100, Biomeda, Foster City, CA), rat anti-mouse Mac-3 antibody (1:100, Pharmingen, San Diego, CA), rabbit polyclonal anti-mouse osteopontin (1:100, ARP, Belmont, MA), rabbit polyclonal anti-CD4 (1:100, Abbiotec, San Diego, CA), rabbit polyclonal anti-CD8 (1:100, ANASPEC, San Jose, CA) or goat polyclonal anti-cathepsin S and anti-cathepsin L (Santa Cruz Biotechnology, Inc., Santa Cruz, CA). Sections stained for cathepsins S and L were blocked with 10% donkey serum in PBS. As negative controls, we used mouse, rabbit, rat or goat IgG at the same concentrations as the corresponding primary antibodies and for cathepsin K and L antibodies tissue sections from cathepsin K- and L-deficient mice were used. Tissue sections were then incubated with the appropriate secondary antibody: goat anti-rabbit Cy3-conjugated, goat anti-mouse Cy2-conjugated antibody (1:200, Rockland, Gilbertsville, PA), goat anti-rat FITC-conjugated antibody (1:200, Sigma-Aldrich, St. Louis, MO), and donkey anti-goat Texas Red-conjugated (1:200 v/v, Jackson ImmunoResearch Laboratories, West Grove, PA).

### Gomori's trichrome staining

Sections were deparaffinized, hydrated with a graded alcohol series and distilled water, and placed in preheated Bouin's solution at 58°C for 10 min. Sections were stained in Weigert's iron hematoxylin solution for 5 min, washed under running water for 30 sec, stained with Gomori's trichrome solution (Sigma-Aldrich, St. Louis, MO) for 15 min, placed in 0.5% acetic acid for 10 sec, and then mounted for light microscopy.

### Morphometry, collagen content, and cell composition of granulomas

The areas of granulomas stained positively for Mac-3, osteopontin, CD4 and CD8, and collagen were quantified using color threshold and calculated as percentage of granuloma area. The size of granulomas was measured in mm^2^. Trichrome stained sections were used for the measurement of granuloma size and for the quantification of collagen content. Images were captured with a Leica DMI 6000B microscope (Leica Microsystems, Inc, Richmond Hill, ON) and analyzed with Openlab 3 software.

### Quantification of the number of granulomas, multinucleated giant cells and CD4-positive cells

Five sections 200 μm apart from each other were analyzed for each lung. For CD4-positive cell quantification three pictures per lung section were taken using a 40-fold magnification, avoiding areas with dominating tracheal or bronchial tissue. The average number of granulomas and multinucleated giant cells per section and the average number of CD4-positive cells per captured area for each mouse were used for statistical analyses.

### Statistical analysis

Student's t-tests were performed when values past test for normal distribution. Data are presented as mean ± standard deviation. Discontinuous data (such as the number of granulomas) were analyzed using the Mann-Whitney U-test. Significance was concluded when P < 0.05.

## Results

### Cathepsins K, L and S are expressed in mouse lung granulomas

**Figure 2:**
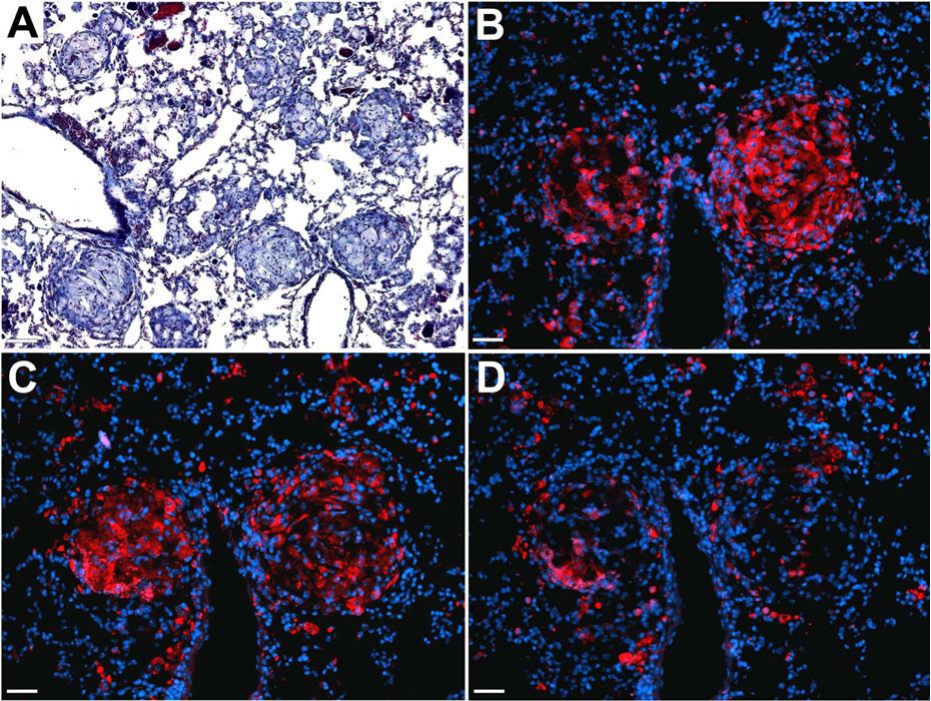
**Expression of cathepsins in lung granulomas**. Cathepsins K, L and S are expressed in lung granulomas of *Apoe-/- *mice. (A), trichrome stained section shows well-formed lung granulomas. (B), cathepsin K immunostaining (red - cathepsin K, blue - nuclei). (C), cathepsin L immunostaining (red - cathepsin L, blue - nuclei). (D), cathepsin S immunostaining (red - cathepsin S, blue - nuclei). (A), scale bar 130 μm, (B-D), scale bars 65 μm.

Recently, we described that *Apoe-/- *mice receiving HFD develop epithelioid lung granulomas with characteristic features of granulomas of sarcoidosis [6]. Granulomas were composed of epithelioid cells, macrophages and T cells, and some granulomas also contained fibroblasts and multinucleated giant cells (MGCs) [6]. Figure 2A displays the typical phenotype of granulomas seen in this model. Similarly to granulomas of sarcoidosis, mouse lung granulomas were intensively stained for cathepsin K with the highest immunoreactivity in epithelioid cells forming the core of the granulomas (Figure 2B). Analysis of consecutive sections stained for cathepsins L and S revealed that similarly to cathepsin K, cathepsin L was prevalently observed in the core of granulomas, whereas cathepsin S staining was weaker and uniformly distributed throughout the lung tissue (Figure 2C, D).

### Cathepsin K deficiency increased the incidence of granulomatous infiltrations in lungs

**Figure 3:**
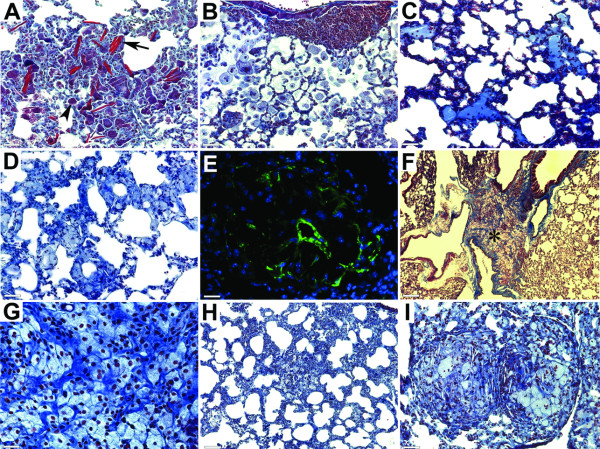
**Structural changes in lungs of *Apoe-/-Ctsk-/- *mice**. (A), extensive fibrosis with Schaumann's bodies (arrowhead) and crystalline structures (arrow). (B), inducible bronchus-associated lymphoid tissues (iBALT) and alveolar monocyte/neutrophilic infiltration. (C), proteinosis. (D), ring-like structures formed by epithelioid cells. (E), smooth muscle cell α-actin positive cells in granulomas (green - smooth muscle cell α-actin, blue - nuclei). (F), accumulation of epithelioid cells between layers of collagen fibers (* shows area magnified on G). (H), individual epithelioid cells in fibrotic areas with honeycombing appearance. (I), two granulomas with relatively large size. (A-D, F-I), trichrome stained sections. (A-E and I), scale bars 65 μm; (F), scale bar 260 μm; (G), scale bar 30 μm; (H), scale bar 130 μm.

Similarly to *Apoe-/- *mice, *Apoe-/- Ctsk-/- *mice receiving HFD showed fibrotic changes in lung tissues and the presence of such inclusions as Schaumann's bodies and crystalline structures (Figure 3A), which are nonspecific but are often present in patients with sarcoidosis. *Apoe-/- Ctsk-/- *mice also developed inducible bronchus-associated lymphoid tissues (iBALT) which are usually induced by inflammatory reactions and are present in bronchovascular bundles [28] (Figure 3B). Evidences of acute lung injury, such as alveolar monocyte/neutrophil infiltration and proteinosis, were also observed (Figure 3B and 3C). In *Apoe-/- *mice, 41% of the animals displayed lung granulomas, whereas in their cathepsin K-deficient littermates this number reached 88% (table 1). Notable differences in the morphology of granulomatous infiltrations were also observed. In some lung areas, the presence of epithelioid cells did not result in the development of well-formed and ball-shaped granulomas that were predominant in cathepsin K-expressing mice. In cathepsin K-knockout mice, epithelioid cells were often organized in amorphous or ring-like structures formed by epithelioid cells (Figure 3D) and granulomas often contained an accumulation of fibroblast-like shaped cells showing positive staining for α-actin (Figure 3E). In some lung areas, extensive accumulation of collagen fibers and epithelioid cells covered large areas between airways and blood vessels (Figure 3F, G). In more fibrotic areas that had honeycombing appearance, individual epithelioid cells were randomly dispersed (Figure 3H). The average size of granulomas was significantly smaller in cathepsin K-deficient mice (see below) when compared to cathepsin K-expressing littermates, but some granulomas reached comparatively large sizes (Figure 2I) that might be related to changes in granuloma development in the absence of cathepsin K.

**Table 1:** Percentage of mice with granulomas, proteinosis or iBALT in lungs

	*Apoe-/-* (16 w)	*Apoe-/-* *Ctsk-/-* (16 w)	*Apoe-/-* *Ctsl-/-* (16 w)	*Apoe-/-* (8 w)	*Apoe-/-* (cat S inh) (8 w)
Granulomas	41	88	31	41	-
Proteinosis	17	11	26	9	8
iBALT	47	77	31	36	15

**Figure 4:**
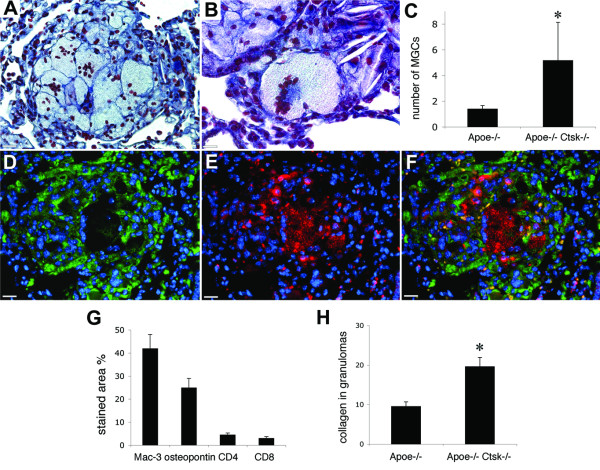
**Granuloma composition in cathepsin K-deficient mice**. (A), granuloma with several foreign body-type MGCs. (B), granuloma with Langhans-type MGC (C), cathepsin K-deficient mice had significantly higher number of MGCs per lung section, (* - p < 0.05 when compared to *Apoe-/- *mice). (D), Mac-3 positive cells in granuloma (green - Mac-3, blue - nuclei), (E), osteopontin-containing epitheloid and giant cells (red - osteopontin, blue - nuclei), (F), merge of D and E. (G), percentage of granuloma area stained for Mac-3, osteopontin, CD4 and CD8. (H) The collagen-stained area was quantified using color threshold and calculated as percentage of granuloma area (* - p < 0.05 when compared to *Apoe-/- *mice). (A, D-F), scale bars 30 μm, (B), scale bar, 20 μm.

Analysis of lung granuloma composition revealed that cathepsin K-deficient mice contained significantly higher number of MGCs per lung section (Figure 4A-C). Granulomas contained foreign body-type MGCs, with random distribution of nuclei (Figure 4A) and Langhans-type MGCs with horseshoe-like arrangements of nuclei (Figure 4B). Epithelioid cells and MGCs were intensively stained for osteopontin, whereas the macrophage marker Mac-3, was not expressed in giant cells (Figure 4D-F). Granuloma cell composition analyzed by immunohistochemical staining did not reveal significant differences in the amount of CD4^+ ^and CD8^+ ^T cells as well as Mac-3 (macrophage marker) and osteopontin positive cells (epithelioid cells marker) in *Apoe-/- Ctsk-/- *mice when compared to their cathepsin K-expressing littermates (Figure 4G and [6]). The collagen content was significantly higher in granulomas of mice lacking cathepsin K (Figure 4H).

### Cathepsins K and L deficiencies and cathepsin S inhibition decreases the number and size of lung granulomas

**Figure 5:**
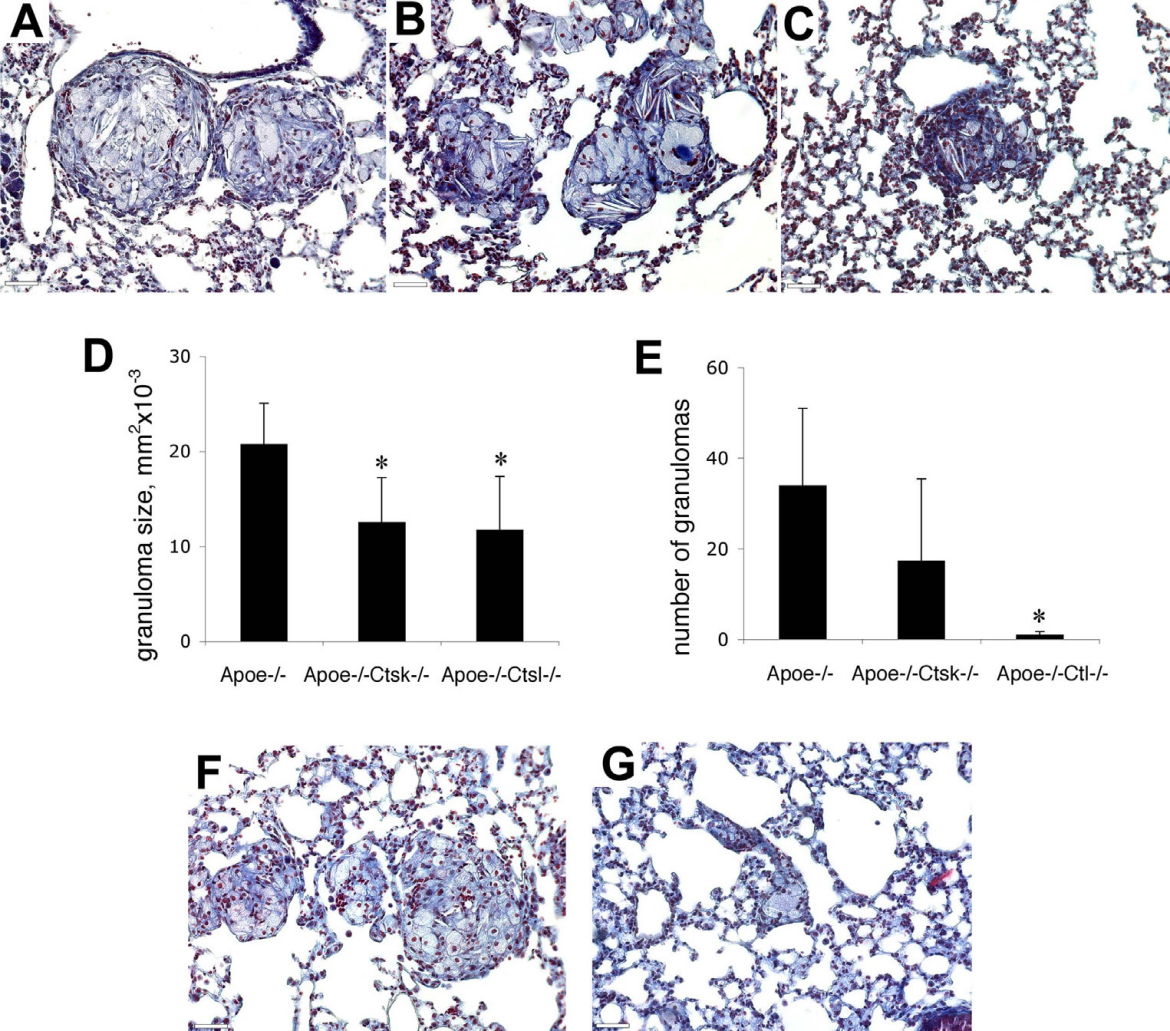
**Changes in granuloma size and numbers**. Granulomas in *Apoe-/- *(A), *Apoe-/-Ctsk-/- *(B) and *Apoe-/-Ctsl-/- *mice after 16 weeks on HFD (C). (D), size of granulomas was significantly smaller in cathepsin K- and L-deficient mice. (E), number of granulomas in cathepsin L-deficient mice was dramatically decreased. (F), granulomas in *Apoe-/- *mice after 8 weeks on HFD. (G), *Apoe-/- *mice treated with cathepsin S inhibitor developed only individual epithelioid cells after 8 weeks on HFD. (A-C, F, G), trichrome stained sections, scale bars 65 μm. * - p < 0.05 when compared to *Apoe-/- *mice.

*Apoe-/- *mice lacking cathepsin K have shown a higher incidence of lung granulomas compared to *Apoe-/- *mice (table 1), but the size of granulomas was significantly smaller (Figure 5A, B, D). Cathepsin L deficiency resulted in the lower incidence and smaller size of granulomas (table 1 and Figure 5C, D). The number of granulomas decreased dramatically from an average of 34 per lung section in *Apoe-/- *mice to one in *Apoe-/- Ctsl-/- *mice (Figure 5E). After 8 weeks of HFD the size of granulomas in *Apoe-/- *mice was smaller compared to 16 weeks of diet (12.6 ± 3.66 mm^2^ vs. 20.8 ± 4.3) but granulomas were still well-formed (Figure 5F) and similarly to 16 weeks, they were present in 41% of mice (table 1). In cathepsin S inhibitor-treated mice only individual epithelioid cells without well-formed granulomas were observed (Figure 5G) and epithelioid cells were present in 30% of mouse lungs. After one week of dosing, morning blood levels of active inhibitor reached 1.0 ± 0.16 μM (n = 6 animals). This concentration likely approximates the peak blood level as mice feed predominantly at night. Cathepsin S is highly expressed in the spleen and antigen-presenting cells and its inhibition impairs antigen presentation and causes a build-up of intermediate invariant chain (Ii) breakdown products [29,30]. Previously, we reported significant accumulation of 10 kDa products of Ii degradation in cathepsin S inhibitor-treated mice after 8 weeks of HFD that provides an evidence of an efficient inhibition of cathepsin S [31].

### Effect of cathepsins on thymus hypertrophy

**Figure 6:**
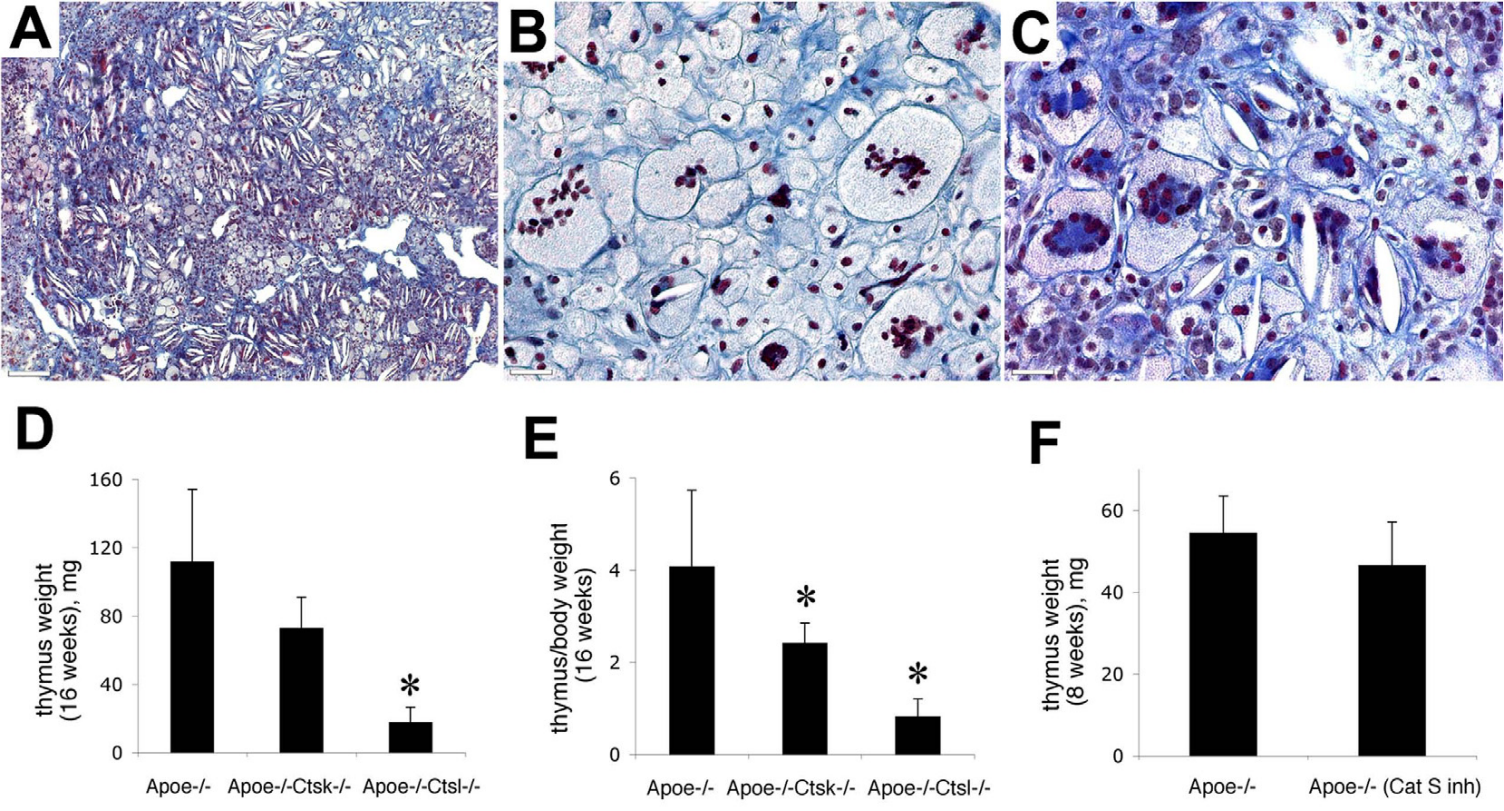
**Fibrosis and MGCs in thymus**. (A), fibrotic thymus in *Apoe-/-Ctsl-/- *mice. (B), foreign body type MGCs with random distribution of nuclei in *Apoe-/-Ctsk-/- *mice. (C), Langhans-type MGCs with horseshoe-like arrangement of nuclei in cathepsin S inhibitor treated mice. (D), cathepsin L deficiency prevents hypertrophy of thymus. (E), proportion of thymus to total body weight was significantly lower in cathepsin K- and L-deficient mice. (F), cathepsin S inhibitor treatment did not prevent thymus hypertrophy. * - p < 0.05 when compared to *Apoe-/- *mice. A-C, trichrome staining. (A), scale bar 130 μm, (B, C), scale bars 30 μm.

In addition to fibrosis and granulomatous inflammation in lung, HFD induces fibrotic changes and hypertrophy in the thymus. Previously, we observed that after 16 weeks on HFD, *Apoe-/- *mice had more than a 3-fold increase in thymus weight compared to their littermates on normal diet [6]. Cathepsin K and L deficiencies and cathepsin S inhibition did not prevent the development of fibrotic changes in thymi. In all mice, an accumulation of collagen fibers and the presence of MGCs were observed (Figure 6A-C). But the thymus hypertrophy was dependent on cathepsin activities: mice receiving the cathepsin S inhibitor, or lacking cathepsin K, have shown insignificantly lower weights of thymi, whereas in *Apoe-/- Ctsl-/- *mice thymi had more than 5 times smaller weights when compared to *Apoe-/- *mice (Figure 6D, F). As cathepsin L-deficient mice had a lower total body weight, a ratio between thymus and total body weight was evaluated. This proportion was significantly lower in cathepsin K-null mice and more than 4-times smaller in cathepsin L-deficient mice (Figure 6E).

### Accumulation of CD4-positive cells in lungs is affected by cathepsin S inhibitor and cathepsin L deficiency

**Figure 7:**
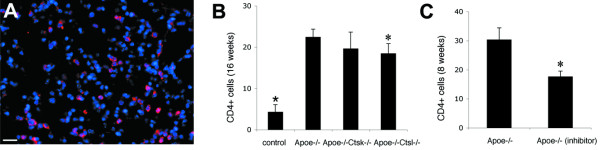
**Effect of cathepsins on the amount of CD4-positive cells in lungs**. The number of CD4-positive cells (A) was more than a 5-times higher in lungs of *Apoe-/- *mice as a result of HFD-feeding (B). *Apoe-/- Ctsk-/- *mice did not reveal a significant difference in its CD4 population, whereas *Apoe-/- Ctsl-/- *mice showed less dramatic accumulation of CD4^+ ^cells when compared to *Apoe-/- mice *(* - p < 0.05 between *Apoe-/- *mice on HFD and *Apoe-/- *mice on normal diet; * - p < 0.05 when compared to *Apoe-/- *mice on HFD). Cathepsin S inhibitor treatment resulted in a significant reduction of CD4^+ ^cells in lungs of mice on HFD (C, * - p < 0.05 when compared to mice on HFD without inhibitor). (A), Scale bar 30 μm.

The formation of lung granulomas was accompanied by a more than 5-fold increase in the number of CD4-positive cells in lungs of mice on HFD when compared to mice on normal diet (Figure 7A, B). Cathepsin K deficiency did not affect the CD4 population when compared to *Apoe-/- mice*. In contrast, disruption of cathepsin L and S activities resulted in an 18% and 43% lower accumulation of CD4-positive cells in lungs (Figure 7B, C).

## Discussion

The formation of lung granulomas results in an extensive extracellular matrix remodeling. Collagen and elastin are the major components of the pulmonary matrix and it is believed that a cooperative action of multiple enzymes is required for its turnover. Cathepsin K has a unique ability to cleave highly efficiently triple helical collagen at multiple sites and to act as a potent elastase as well [32]. Its strong expression in lung granulomas suggested an important role in granuloma formation and resolution, and indeed, lungs of *Apoe-/- Ctsk-/- *mice showed significant differences in granuloma appearance, size, and composition. The increased incidence of lung granulomas in cathepsin K-deficient mice (88% vs. 41%) correlates with the increased collagen fibers deposition (10% vs 20%) that may consequently result in a delayed granuloma resolution. Lung granuloma formation and their spontaneous resolution as commonly observed in patients with sarcoidosis [33] require remodeling of the extracellular matrix. It is tempting to speculate that the increased amount of collagen in granulomas (Figure 4H) and the presence of abundant collagen fibers between epithelioid cells in areas without well-formed granulomas (Figure 3F, G) in *Ctsk-/- *mice is due to the lack of the collagenase activity of cathepsin K. This accumulation of collagen fibers might also be responsible for the smaller number and size of granulomas. In our measurements, accumulations of epithelioid cells were considered as granulomas only when they were present as well-formed and ball-shaped structures that are characteristic for sarcoidosis. In *Apoe-/- Ctsk-/- *mice, epithelioid cells were often observed forming ring-like structures with dispersed collagen fibers, or were randomly distributed in fibrotic areas (Figure 3D, H).

The cell composition of granulomas in cathepsin K-deficient mice was also changed. They contained a significantly higher number of MGCs. This type of cells is formed by the fusion of cells from the monocyte-macrophage lineage [34]. In atherosclerotic plaques from *Apoe-/- Ctsk-/- *mice, macrophages have an increased size [35] and microarray analyses have revealed the upregulation of several macrophage genes, including CD36 [36]. Since CD36 participates in macrophage fusion [37], we suggest that it might be responsible for the increased number of MGCs in cathepsin K-deficient mice. The disruption of cathepsins L and S activities resulted in a more significant effect on granuloma formation. Cathepsin L deficiency reduced the size and the number of lung granulomas whereas mice treated with the cathepsin S inhibitor did not develop well-formed granulomas.

Analysis of available literature data suggests that the preventive effect of cathepsin L deficiency or cathepsin S inhibition on lung granuloma development may not only depend on their extracellular matrix-degrading activities, but on their involvement in antigen presentation as well [17,38]. Cathepsin S is expressed in B cells, macrophages, and dendritic cells and is required for invariant chain (li) degradation and antigen processing [30]. Cathepsin S-deficient mice have decreased MHC class II presentation in B and dendritic cells, and a reduced number of CD4^+ ^T-cells [15]. On the other hand, cathepsin L is expressed in cortical thymic epithelial cells and macrophages and is responsible for li degradation in CD4^+ ^T cell selection in the thymus [17]. Cathepsin L-deficient mice have a reduction in their numbers of CD4^+ ^cells in thymus and peripheral organs [39]. The roles of cathepsins S and L on the functions of CD4^+ ^may have a direct influence on sarcoidosis development. T cell activation is mandatory for the development of granulomatous reactions and CD4^+ ^T cells of the Th1-type are essential for the formation and the maintenance of granulomas [18-21]. The decreased amount of CD4^+ ^cells in lungs of mice lacking cathepsin L or treated with cathepsin S inhibitor (Figure 7B, C) supports the suggestion that the effect of these two cathepsins on the granuloma formation is related to their role in antigen presentation and T-cell selection.

In addition to its involvement in antigen presentation, cathepsin L has been described to play a role in T-cell actin polymerization, shape polarization, chemotaxis. Cathepsin L deficiency significantly decreases the expression of laminin, fibronectin, and collagens I and II in thymus of cathepsin L-deficient mice [40]. This might partially explain why *Apoe-/- Ctsl-/- *mice had significantly smaller thymi compared to *Apoe-/- *mice (Figure 7D, E).

In conclusion, our results show that the disruption of cathepsin L and S activities prevents the development of lung granulomas, whereas cathepsin K deficiency results in altered granuloma composition in mice. These results suggest that interventions in cathepsin S and L activities may yield a therapeutic benefit for patients with sarcoidosis, i.e., a potential prevention of disease progression. Such an approach, however, would require extreme caution as both cathepsins have been described as critical participants in antigen presentation and their inhibition may result in increased infection and cancer rates.

## Declaration of competing interests

The authors declare that they have no competing interests.

## Statement of Authors' contributions

AOS designed and carried out experiments, analysed data and wrote the paper, JYG carried out experiments with cathepsin S inhibitor, MDP designed and carried out experiments with cathepsin S inhibitor and wrote inhibitor-related sections of the paper, DB conceived experiments, analysed data and wrote the paper. All authors have approved the final manuscript.
